# Cognitive Deficit-Related Interhemispheric Asynchrony within the Medial Hub of the Default Mode Network Aids in Classifying the Hyperthyroid Patients

**DOI:** 10.1155/2018/9023604

**Published:** 2018-11-08

**Authors:** Mengmeng Zhi, Zhenghua Hou, Yuqun Zhang, Yingying Yue, Ling Li, Yonggui Yuan

**Affiliations:** ^1^Department of Endocrinology, Affiliated Zhongda Hospital, School of Medicine, Southeast University, Nanjing 210009, China; ^2^Department of Psychosomatics and Psychiatry, Affiliated Zhongda Hospital, School of Medicine, Southeast University, Nanjing 210009, China; ^3^Department of Psychiatry, Columbia University College of Physicians and Surgeons, The New York State Psychiatric Institute, New York, NY 10032, USA; ^4^Institute of Psychosomatics, School of Medicine, Southeast University, Nanjing 210009, China

## Abstract

**Background and Purpose:**

Recent studies suggest that abnormal structure and function in the brain network were related to cognitive and emotional impairment in hyperthyroid patients (HPs). The association between altered voxel-mirrored homotopic connectivity (VMHC) and neuropsychological impairment in HPs remains unclear. This study is aimed at investigating the association between the disrupted functional coordination and psychological dysfunction in hyperthyroidism.

**Method:**

Thirty-three hyperthyroid patients and thirty-three matched healthy controls (HCs) were recruited, and they received resting-state functional magnetic resonance imaging (fMRI) scans and neuropsychological evaluation. The VMHC value was computed to reveal the functional coordination between homotopic regions in both groups. The neurobehavioral relevancy method was employed to explore the relationship between the altered VMHC and emotional, cognition measures. Further receiver operating characteristic (ROC) curve analysis was adopted to examine the power of changed regional VMHC in classifying the patients with hyperthyroidism.

**Results:**

Compared with the HCs, the HPs exhibited significantly declined VMHC values in the bilateral medial frontal gyrus (MeFG). The interhemispheric asynchrony in the MeFG was positively correlated with *Z* scores of episodic memory. The ROC analysis further determined that abnormal VMHC in the MeFG could efficiently distinguish the HPs from the HCs (area under the curve (AUC) = 0.808, *P* < 0.001).

**Conclusion:**

The altered interhemispheric coordination in the hub of the default mode network may implicated in the modulation of episodic memory in HPs patients and the distinct feature of the interhemispheric asynchrony may be treated as a potential target for the early recognition and intervention for the HPs with cognitive impairments.

**Clinical Trial Registration:**

This is a study of the neurological basis for dysfunction of mood and cognition in hyperthyroid patients: a resting-state fMRI study (registration number: ChiCTR-OOC-16008607).

## 1. Introduction

Thyroid hormones have been reported to contribute to brain development through neurogenesis, development of glia, myelination, synaptogenesis, and dendritic proliferation [1–3]. Deficiency of thyroid hormone in adults can cause cognitive impairment in a variety of cognitive domains, even dementia [4–6]. To date, hyperthyroidism, which refers to excessive circulating thyroid hormones, is associated with a range of specific neuropsychological impairments such as nervousness, irritability, tremulousness, depression, anxiety, lack of concentration, and cognitive dysfunction [7–11]. On account of this, it is urgent to explore the potential neural substrate of impaired emotion and cognition in hyperthyroidism.

Morphological, functional, and metabolic brain alterations in hyperthyroidism have been revealed to be correlated with the changes in mood and cognition, and the related brain regions are mainly located in the default mode network (DMN). Zhang et al. [12] reported that the atrophy gray matter volume (GMV) of the bilateral hippocampus was negatively correlated with the severity of disease in patients with HPs. Inconsistently, another voxel-based morphometry study observed greater GMV in the right posterior lobe of the cerebellum and reduced GMV in the bilateral visual cortex and anterior cerebellum in HPs relative to euthyroid subjects, and the altered GMV was positively related to the performance of sensorimotor functions and working memory [13]. In addition to anatomic brain variations, resting-state functional magnetic resonance imaging (rs-fMRI) studies further confirmed aberrant functional connectivity in patients with HPs. A significantly disrupted functional connectivity between the hippocampus and bilateral anterior and posterior cingulate cortex (ACC and PCC) and right medial orbitofrontal cortex was found [14], and there was a negative correlation between the altered functional connectivity and severity of depression and anxiety in the HP group. Furthermore, an enhanced functional connectivity exists between temporal poles and DMN regions, including the precuneus, inferior parietal lobule, medial frontal cortex, cingulate cortex, ventromedial orbitofrontal cortex, and hippocampus in drug-induced hyperthyroid patients [15]. Our previous study also revealed similar brain regions with altered functional connectivity in the HPs, and the impaired connectivity was positively related to the dysfunction of emotion and cognition [16]. Regarding metabolic researches, patients with HPs were proven to have reduced glucose metabolism in the frontal lobe, temporal lobe, and parahippocampal gyrus compared with HCs, and the activation foci in the PCC and inferior parietal lobe were correlated with the severity of anxiety and depression [17, 18]. Meanwhile, two additional studies demonstrated a prominently decreased concentration of glutamate in the PCC in the HP group, indicating an underlying role of glutamate in the processing of brain dysfunction [19, 20]. Above all, the above evidence converges at one point that the abnormal regions involved in HPs were mainly located in the DMN, and the functional alterations were deeply implicated in dysregulation of neuropsychological function. Therefore, it is reasonable to speculate whether the impairment of the DMN can constitute the neural substrates of emotional and cognitive dysfunction in HP patients.

In spite of the amalgam of research collected so far, hyperthyroidism-related changes in the functional or structural interactions between cerebral hemispheres have been directly examined on no occasions. Functional homotopy, defined as a high degree of synchrony in spontaneous activity between geometrically symmetrical interhemispheric regions, is one of the most prominent attributes of the brain intrinsic architecture [21]. It considered the process of interhemispheric communication to integrated brain function underlying coherent cognition and behavior [22, 23]. A novel measure called voxel-mirrored homotopic connectivity (VMHC) reflects the resting-state functional synchrony between each voxel in one hemisphere and its opposite counterpart in the mirrored hemisphere [24, 25]. Alterations in the VMHC have been discovered in depression [26], schizophrenia [27], chronic tinnitus [28], psychiatric disorders [29], and substance dependence [24], which suggests that VMHC may be a sensitive tool to detect the alterations of interhemispheric coordination in both normal aging and disease states. The purpose of the present study was to characterize the intrinsic difference of the interhemispheric coordination between HP patients and HCs, so as to explore the underlying mechanism of neuropsychological impairments in hyperthyroidism.

## 2. Materials and Methods

### 2.1. Participants

A total of thirty-three right-handed hyperthyroid patients and thirty-three age-, sex-, and education-matched healthy controls were recruited (age range: 18-60 years, education range: 9-22 years). All the patients had elevated serum FT3, FT4, and thyrotropin receptor antibody (TRAb) levels and inhibited TSH levels. However, the thyroid hormones of the control group were within normal ranges (FT3 1.8-4.6 pg/mL, FT4 0.93-1.7 ng/dL, TRAb 0-1.75 IU/L, and TSH 0.27-4.2 *μ*IU/mL). All the patients and controls received MRI scanning as well as neuropsychological assessments. The exclusion criteria and neuropsychological tests can be seen in our previous study [16]. The thyroid hormone levels, disease duration, height, weight, and family history were recorded in this research. This study was approved by the Medical Ethics Committee for Clinical Research of Zhongda Hospital Affiliated to Southeast University. All the participants signed written informed consent prior to the study.

### 2.2. MRI Data Acquisition and Data Analysis

#### 2.2.1. MRI Data Acquisition

All imaging data were acquired using a 3.0 T MRI scanner (Siemens MAGNETOM Trio, Erlangen, Germany) with a standard head coil. In order to reduce the head motion and scanner noise during scanning, all subjects were instructed to lie quietly with the head fixed by a belt and ears covered with foam padding and earplugs. Meanwhile, they were required to close their eyes and keep awake, and any specific thoughts were avoided during the scan. Resting-state images were acquired using a gradient-echo planar sequence with the following scan parameters: repetition time = 2000 ms, echo time = 25 ms, flip angle = 90°, acquisition matrix = 64 × 64, field of view = 240 mm × 240 mm, thickness = 3.0 mm, slices = 36, gap = 0 mm, and 3.75 mm × 3.75 mm in-plane resolution parallel to the anterior commissure–posterior commissure line. For each participant, rs-fMRI scanning lasted 8 minutes and 240 volumes were obtained.

#### 2.2.2. Image Preprocessing

Functional images were preprocessed with the Data Processing Assistant for Resting-State Function (DPARSF 2.3 advanced edition) MRI toolkit [30], which integrates procedures based on the Resting-State Functional MRI toolkit (REST, http://www.restfmri.net) [31], and statistical parametric mapping software package (SPM8, http://www.fil.ion.ucl.ac.uk/spm). The first 10 time points were removed to ensure stable-state longitudinal magnetization and adaptation to inherent scanner noise. The remaining 230 resting-state fMRI images were sequentially performed according to the following steps: (1) slice timing with the 35th slice as reference slice, corrected for temporal differences and head motion correction (participants with head motion of greater than 1.5 mm of maximum displacement in any direction (*x*, *y*, or *z*) or 1.5 degrees of angular motion were excluded from the present study); (2) core registering T1 to functional image and then reorienting; (3) for spatial normalization, segmenting T1-weighted images into white matter, gray matter, and cerebrospinal fluid, which are then normalized to the Montreal Neurological Institute space by using a 12-parameter nonlinear transformation (the above transformation parameters were performed to the functional images, and then the functional images were resampled with isotropic voxels of 3 mm); (4) adopting a 6 mm full-width at half-maximum isotropic Gaussian kernel for spatial smoothing; (5) detrending the linear trend within each voxel's time series; (6) regressing out nuisance signals (white matter, cerebrospinal fluid signals, and head-motion parameters calculated by rigid body 6 correction) and spike regressors; and (7) minimizing the low-frequency drift and high-frequency noise filtered with a temporal bandpass (0.01-0.08 Hz).

#### 2.2.3. Voxel-Mirrored Homotopic Connectivity

For the calculation of the VMHC in the geometric configuration of the bilateral hemispheres, the preprocessed functional images were transformed to the symmetric space with the following procedure: (a) generating a mean image by averaging the normalized gray matter images for all participants, (b) averaging the mean image with a bilateral mirrored version to create a symmetrical template with group specificity, and (c) registering every individual normalized gray matter image to the generated symmetric template and then transforming for functional images by the nonlinear strategy. The unilateral hemispheric templates of the symmetric gray matter were used as a mask for individual-level computation of the VMHC. Then, Pearson's correlation analysis was adopted between each voxel and the mirrored counterpart voxels within the interhemispheric symmetry in each subject. The correlation coefficients were then normalized to a *z*-map with the Fisher *z*-transformation. The above procedures were performed utilizing the DPARSF 2.3 software. Finally, the acquired resultant values constituted the VMHC measures for statistical analysis. The threshold of statistical maps were set at *P* < 0.05 and corrected by the 3dClustSim program in the AFNI software (https://afni.nimh.nih.gov/pub/dist/doc/program_help/3dClustSim.html). The details of the VMHC acquisition have been elucidated in a previous study [25].

#### 2.2.4. Statistics Analysis

The differences in demographic and neuropsychological performances between hyperthyroid patients and healthy controls were determined by various statistical methods. Two-sample *t*-tests and Mann-Whitney rank tests were applied for continuous variables, and the chi-square test was applied for categorical variables (statistical significance was set at *P* < 0.05) by SPSS 21.0 software (SPSS Inc., Chicago, IL).

We separated the cognitive tests into 4 domains as above (i.e., processing speed, executive function, visuospatial skills, and episodic memory). To combine the cognitive variables, the standardized *Z* scores of each individual test, which were created by using control group data across all patients, were further summed to figure out the cognitive domain values. Variables in which good performance was represented by lower values (e.g., TMT, Stroop Color and Stroop Word) were adjusted for reciprocal transformation to ensure that higher *Z*-scores represented better performance for all variables. The independent sample *t*-test was performed to compare the mean *Z*-scores for each neuropsychological test and cognitive domain in order to compare the patterns of neurocognitive impairments between the two subgroups.

To investigate significant differences of the regional VMHC between the HPs and the HCs, a two-sample *t*-test after correction with the 3dClustSim program (*P* < 0.01, cluster sizes > 85 voxels) was performed by AFNI software (https://afni.nimh.nih.gov/pub/dist/doc/program_help/3dClustSim.html). Within the hyperthyroidism group, to detect the regional VMHC that was significantly correlated with the neuropsychological performance in the patients, a voxel-wise general linear model was adopted between the zVMHC maps and the standardized neuropsychological performance scores [32]. The statistical threshold was defined at *P* < 0.05, correcting for multiple comparison by 3dClustSim with age, education, and gender as covariates in the model. In line with our previous study [26], we determine the predictive power of the altered VMHC values with receiver operating characteristic (ROC) curves (area under curve (AUC): 0.9-1.0 = excellent, 0.8-0.9 = well, 0.7-0.8 = fair, 0.6-0.7 = poor, and 0.5-0.6 = fail), sensitivity, and specificity. The optimal cut-off point was determined by the corresponding maximized Youden's index *J* (*J* = sensitivity + specificity − 1). Binary logistic regression analysis was employed to integrate the combined discrimination capacity of changed VMHC. The threshold of statistical significance was defined as *P* < 0.05.

## 3. Results

### 3.1. Demographic and Neuropsychological Results

There were no significant between-group differences in age, education, and gender regarding the demographics and neuropsychological data. The FT3, thyroid peroxidase antibody, and thyroglobulin antibody levels of hyperthyroid patients were significantly higher than those of HCs (*P* < 0.001). Compared with the HC group, the hyperthyroid group showed significantly higher scores in the HDRS (*P* < 0.001) and HARS (*P* < 0.001), but lower scores in executive function (*P* = 0.011) and visuospatial skills (*P* < 0.001). All the specific figures were presented in our previous study [16].

### 3.2. Voxel-Mirrored Homotopic Connectivity Data

**Figure 1:**
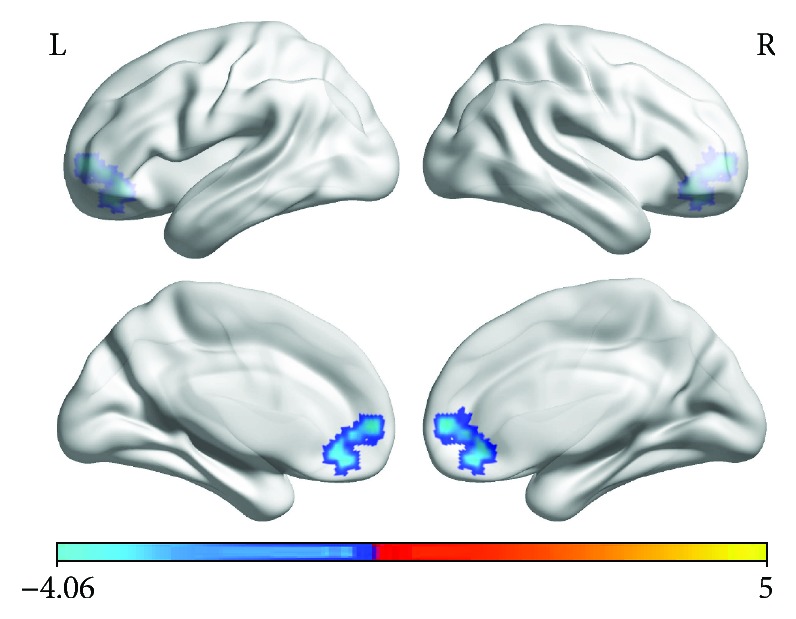
Significantly decreased (blue) VMHC in hyperthyroid patients relative to healthy controls. (*P* < 0.01, 3dClustSim correlated). The color bar indicates the *T* value from the two-sample *t* test between the hyperthyroidism and healthy control groups.

Compared with the healthy subjects, the hyperthyroidism group showed significantly decreased VMHC values in the bilateral medial frontal gyrus (MeFG) (Table 1, Figure 1).

### 3.3. Correlation Analysis within the Hyperthyroidism Group

**Figure 2:**
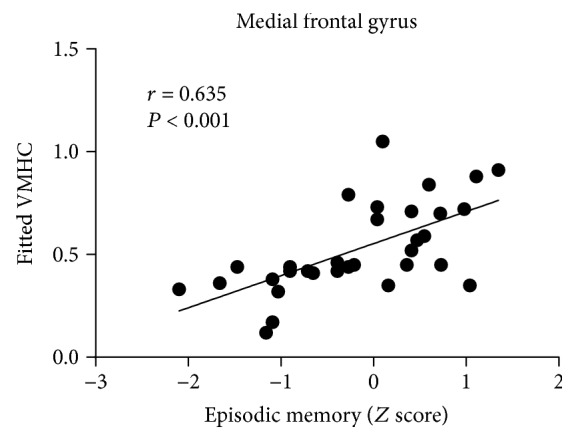
Scatter diagram shows the correlation between the psychological assessment and the VMHC values in the hyperthyroidism group. The VMHC in the MeFG was positively correlated with episodic memory (*r* = 0.635, *P* < 0.001). VMHC: voxel-mirrored homotopic connectivity; MeFG: medial frontal gyrus; *r*: Spearman's correlation coefficient.

After controlling for age, gender, and education level, partial correlation analyses were applied within the hyperthyroidism group. The interhemispheric asynchrony in the MeFG was positively correlated with *Z* scores of episodic memory (Figure 2).

### 3.4. ROC Analysis between Hyperthyroidism Group and Control Group

**Figure 3:**
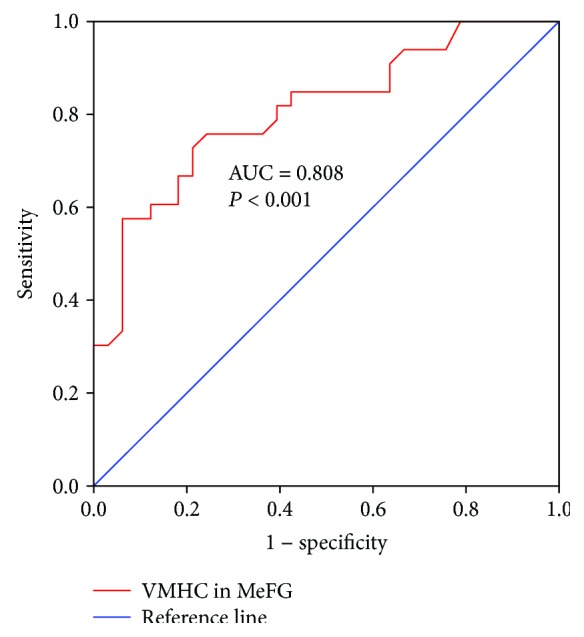
The diagnostic performance of the disrupted VMHC in classifying the HPs from the HCs. VMHC: voxel-mirrored homotopic connectivity; HPs: hyperthyroid patients; HCs: healthy controls; MeFG: medial frontal gyrus; AUC: area under the curve.

The ROC analysis demonstrated that the regional VMHC changes of MeFG (AUC = 0.808, *P* < 0.001) exhibited good performance in distinguishing HP patients from healthy controls, with sensitivity (75.8%) and specificity (75.8%) (Figure 3).

## 4. Discussion

To our knowledge, this is the first study to adopt the VMHC to identify changes in interhemispheric functional connectivity and associate these alterations with disrupted emotion and cognition in hyperthyroid patients. The primary finding of this work is the hyperthyroidism-related reduction of brain homotopic connectivity in the hyperthyroid group. As compared with the HC group, the HP group exhibited significant reduction of VMHC in the bilateral MeFG. Furthermore, the VMHC in MeFG was correlated with the impaired episodic memory in hyperthyroidism. Finally, in particular, significant decreases in the VMHC of the MeFG could provide the ability to differentiate hyperthyroid patients from healthy subjects.

The MeFG is a crucial node of the DMN, which is substantially involved in the emotional and cognitive processing, including the process of working memory [33, 34], learning function [35], episodic memory [36], and coordinating self-referential cognitive operations [37, 38]. Our previous study found significant decreases in functional connectivity, regional homogeneity, and amplitude of low-frequency fluctuation in the MeFG [16], and the abnormal functional connectivity was correlated with thyroid hormone levels, anxiety severity, and processing speed of hyperthyroid patients, suggesting the important role of the MeFG in regulating impaired cognition in hyperthyroidism. The reduced interhemispheric functional connectivity in the MeFG of hyperthyroid patients, as well as the positive correlation between the VMHC in the MeFG and episodic memory, further proved that abnormal brain function in the medial structure of DMN was associated with aberrant cognitive function in hyperthyroidism.

Importantly, the noticeable effect of the changed regional VMHC in the MeFG on differentiating hyperthyroidism was also primarily confirmed. In the present study, the ROC curve indicated that the VMHC change of the MeFG in the DMN can effectively distinguish the hyperthyroid patients from HCs. According to the results of the present study, the MeFG in DMN [39, 40] may be involved in some neurobehavioral dysfunctions in hyperthyroidism. This finding is consistent with the results of some former studies which indicated functional, structural, and metabolic brain changes in the DMN. Zhang et al. [14] found abnormalities in functional connectivity within DMN between the hippocampus and both posterior cingulate cortex and medial orbitofrontal cortex, and the disrupted functional connectivity strength was negatively related to the disease duration. Another research indicated that the temporal pole is strongly connected to brain regions comprising the DMN resting-state network, including the precuneus, inferior parietal lobule and medial frontal cortex, cingulate cortex, ventromedial orbitofrontal cortex, and hippocampus [15]. In addition, reduced GMV in the bilateral hippocampus and parahippocampal gyrus of DMN has been observed in a voxel-based morphometry study [12]. The positron emission tomography survey illustrated that hyperthyroid patients exhibited lowered brain glucose metabolism in the DMN regions of the frontal lobe and temporal lobe, and the severity of depression and anxiety covaried negatively with metabolic activity in the inferior temporal and inferior parietal gyri, respectively [17]. Our previous investigation on hyperthyroidism also located the brain regions with impaired functional connectivity in the DMN, embracing the medial frontal lobe, middle temporal gyrus, and precuneus, and the functional connectivity in the precuneus showed a negative correlation with processing speed [16]. All the integrative results from the current and preexisting studies suggest that the DMN possibly plays a pivotal role in compensating the dysregulation of the emotional and cognitive dysfunction in hyperthyroidism. In addition to the DMN, our previous research found that abnormal functional connectivity in the attention network, visual network, and cognitive network possibly constituted the latent mechanism for emotional and cognitive changes in hyperthyroidism. However, the potential association between these networks remains unknown; for this purpose, some novel methods, such as the independent component analysis, can be adopted to reveal the interactions between these networks in future works.

This study also has a few limitations. Firstly, this is a cross-sectional study on whether the altered functional connectivity is reversible after therapy remains to be discussed by the prospective study. Secondly, the trier being unable to control the participants' thoughts during imaging is a common problem to resting-state studies. Although participants were instructed not to move their heads and to rest with their eyes closed, slight head movements and rotation are unavoidable. However, we have inspected each image carefully, and patients with head movements greater than 1.5 or 1.5 mm were excluded. Finally, on account of laboratory testing limits in the hospital, exact figures of FT4, TSH, and TRAb were inapplicable, so we could not compare the variance of brain activation in hyperthyroidism of different severities. Given these limitations, future studies should be well designed, taking these results into consideration. Future fMRI studies could investigate these patients after recovery from hyperthyroidism and to explore the potential brain difference between hyperthyroidism with and without emotional impairment.

## 5. Conclusion

The decreased interhemispheric synchrony in the MeFG anchored in the DMN possibly constitutes the underlying mechanism for neuropsychological changes in hyperthyroidism. The findings of this study imply that the interhemispheric connectivity in the DMN may compensate the neurobiological mechanism of cognitive impairments in hyperthyroid patients. Moreover, the altered interhemispheric coordination in the hub of the default mode network may be implicated in the modulation of episodic memory in HP patients, and the distinct feature of the interhemispheric asynchrony in the MeFG may be treated as a potential target for the early recognition and intervention for the HPs with cognitive impairments.

## Figures and Tables

**Table 1:** Brain regions showing significantly different VMHC between groups.

	Brain regions	BA	MNI coordinates			Voxel number	Peak *t* value
			*X*	*Y*	*Z*		
HPs < HCs	MeFG	32	6	54	0	90	-4.06

Note: A corrected threshold of *P* < 0.01 corrected by 3dClustSim; cluster size is in mm^3^; two-sample *t* tests with age, gender, and education level as covariates were performed to test the VMHC differences between groups. MNI: Montreal Neurological Institute space; HPs: hyperthyroid patients; HCs: health controls; BA: Brodmann area; MeFG: medial frontal gyrus.

## Data Availability

The data used to support the findings of this study are available from the corresponding author upon request.

## Supplementary Materials

Supplementary Materials Supplementary data include the exclusion criteria and neuropsychological tests that we used in this study. Besides, demographic and clinical characteristics as well as mood and cognitive performances are also presented.
