# Supplementary material for: Cognitive Deficit-Related Interhemispheric Asynchrony within the Medial Hub of the Default Mode Network Aids in Classifying the Hyperthyroid Patients
Source: Neural Plast. 2018 Nov 8;2018:9023604. doi: 10.1155/2018/9023604 (PMC6250039; doi:10.1155/2018/9023604)
Supplement: Supplementary Materials — Supplementary data include the exclusion criteria and neuropsychological tests that we used in this study. Besides, demographic and clinical characteristics as well as mood and cognitive performances are also presented. [file 9023604.f1.doc]

**Cognitive deficit related interhemispheric a****synchrony within the medial hub of default mode network** **aid in classifying the hyperthyroid patients**

In order to fit the double-blinded review, we have deleted the reference of our previous study, however in the study, we have cited some materials of the previous study. Here we presented the materials to promote the review.

**The exclusion criteria and neuropsychological tests in our previous study can be seen below:**

Exclusion criteria for all the participants were as follows: (1) A history of neurological or psychiatric illness, including head injury, cerebral hemorrhage, major depression and so on; (2) Drug or alcohol abuse history; (3) A history of cardiovascular or pulmonary diseases that affected BOLD fluctuations; (4) Contraindications to MRI scanning; (5) Head motion more than 1.0 mm or 1.0 u during MRI scanning. The thyroid hormone levels, disease duration, height, weight and family history were recorded in this research.

A [series](../../../../D:%5CUsers%5CBathery%5CAppData%5CLocal%5CYoudao%5CDictBeta%5C7.0.0.1111%5Cresultui%5Cdict%5Cresult.html%3Fkeyword=series) of psychometric and domain-specific cognitive tests were conducted for each subject. The mood tests included Hamilton depression rating scale 17 (HDRS-17) and the Hamilton anxiety rating Scale 17 (HARS-17). The domains of cognition tests consisted of processing speed, executive function, visuospatial skills and episodic memory. Processing speed was evaluated by portions of the Trail Making Test (TMT A), Digit Symbol Substitution Test (DSST), the Stroop Color and Word Test (Stroop Color and Stroop Word). Executive function was assessed via Verbal Fluency Test (VFT-animal, VFT-verb), the Stroop Color Word Test (Stroop Inhibition), TMT B and Digit Span Test (DST). Visuospatial Skills were estimated with Rey-Osterrieth Complex Test (CFT) and Clock Drawing Test (CDT). Episodic memory was assessed with delayed recall of Auditory Verbal Learning Test (AVLT-DR) and Rey-Osterrieth Complex Test (CFT-DR). Two board-certiﬁed clinical psychiatrists scored these tests.

**All the specific figures in our previous study were presented as below:**

Table Demographic, clinical characteristics，mood and cognitive performances

| Characteristic | Hyperthyroidism | Control | p-value |
| --- | --- | --- | --- |
| n  Age (years) | 33  37.36±12.43 | 33  39.03±13.28 | 0.600^a^ |
| Female, n (%) | 26.00(78.79) | 26.00(78.79) | 1.000^b^ |
| Education levels (years) | 13.24±3.82 | 13.70±4.06 | 0.641^a^ |
| BMI (kg/m^2^)  FT3 (pg/ml)  TGAb (IU/mL)  TPOAb (IU/mL) | 21.61±2.81  14.17(8.98-23.59)  269.00(25.07-540.60)  221.20(37.52-493.10) | 23.35±2.55  3.02(2.77-3.33)  18.95(16.10-37.28)  14.84(11.75-19.29) | 0.011^a^  <0.001^c^  <0.001^c^  <0.001^c^ |
| Drug use, n (%) | 15.00(45.45) | - | - |
| Disease duration (months)  Neuropsychological test data (z-score) | 9.94±17.31 | - | - |
| Mood  HDRS^d^  HARS^d^ | 0.70±0.97  0.78±0.85 | -0.70±0.25  -0.78±0.23 | <0.001^a^  <0.001^a^ |
| Processing Speed | -0.17±0.89 | 0.17±0.82 | 0.115^a^ |
| Stroop Color^d^ | -0.15±1.09 | 0.15±0.89 | 0.216^a^ |
| Stroop Word^d^ | -0.17±1.02 | 0.17±0.97 | 0.174^a^ |
| TMT-A^d^ | -0.18±1.00 | 0.18±0.98 | 0.140^a^ |
| DSST^d^ | -0.17±1.00 | 0.17±0.98 | 0.169^a^ |
| Executive Function | -0.24±0.76 | 0.24±0.71 | 0.011^a^ |
| Stroop Inhibition^d^  TMT-B^d^  DST^d^  VFT-1^d^  VFT-2^d^  Visuospatial Skills  CFT^d^  CDT^d^  Episodic Memory  AVLT-DR^d^  CFT-DR^d^ | -0.03±0.97  -0.25±0.81  -0.30±0.99  -0.34±1.02  -0.27±1.13  -0.42±0.92  -0.33±1.27  -0.51±0.97  -0.19±0.87  -0.06±0.97  -0.31±1.02 | 0.03±1.04  0.25±1.12  0.30±0.93  0.34±0.87  0.27±0.78  0.42±0.43  0.33±0.44  0.51±0.74  0.19±0.86  0.06±1.04  0.31±0.89 | 0.807^a^  0.042^a^  0.014^a^  0.005^a^  0.026^a^  <0.001^a^  0.007^a^  <0.001^a^  0.087^a^  0.611^a^  0.011^a^ |

Abbreviations: BMI = Body Mass Index, FT3 = Free Triiodothyronine, TGAb = Thyroglobulin Antibody, TPOAb = Thyroid Peroxidase Antibody, HDRS = Hamilton Depression Rating Scale, HARS = Hamilton Anxiety Rating Scale, Stroop Color Total Time = Color naming subtest scaled score, Stroop Word Total Time = Word naming subtest scaled score, Stroop Inhibition Time = inhibition subtest scaled score; TMT = Trail-Making Test, DSST = Digit Symbol Substitution Test, DST = digit span test, VFT =Verbal Fluency Test, CDT = clock-drawing test, CFT = Rey-Osterrieth complex figure test, CFT-DR = Rey-Osterrieth complex figure test delayed recall, AVLT-DR = auditory verbal learning test–delayed recall.

^a^ Two independent sample t-test.

^b^ Chi-square test.

^c^ Mann-Whitney rank test.

^d^ Scale scores were transformed to standard Z values in order to avoid the inﬂuence of the different measurement units.
